# Case Report: A rare case of acute herbicide poisoning induced pediatric acute osteofascial compartment syndrome

**DOI:** 10.3389/fped.2025.1603429

**Published:** 2025-09-17

**Authors:** Xueping Shi, Xin Wang, Pei Guo

**Affiliations:** ^1^ College of Medical Technology, Chongqing Medical and Pharmaceutical College, Chongqing, China; ^2^ Intensive Care Unit, The First Affiliated Hospital of Chongqing Medical and Pharmaceutical College, Chongqing, China; ^3^ Department of Pharmacy, The First Affiliated Hospital of Chongqing Medical and Pharmaceutical College, Chongqing, China

**Keywords:** pediatric acute osteofascial compartment syndrome, herbicide poisoning, diquat, decompressive surgical fasciotomy, acute osteofascial compartment syndrome

## Abstract

Acute osteofascial compartment syndrome (AOCS) is rarely seen in patients with acute herbicide poisoning, and no cases have been reported in children. The correct treatment approach is of vital importance. We report a case of a 13-year-old girl who was admitted to our hospital 17 h after oral ingestion of “diquat” (a herbicide), and developed clinical symptoms of AOCS 21 h later. The girl was treated with measures such as removing the toxins, using mannitol for dehydration, and elevating the affected limb. The patient's AOCS progressed relentlessly; on hospital day 4, after failure of conservative management, she underwent decompressive surgical fasciotomy (DSF) of the lower legs. The girl responded well to continuous renal replacement therapy (CRRT) and continuous fascial decompression treatment. On hospital day 25, she underwent debridement and suture surgery on both lower legs. At discharge, the girl's lower legs bilateral suture incisions were healing well, and the affected legs could stand. Two months later, the girl was able to walk independently.

## 1 Introduction

AOCS is a serious complication of traumatic orthopedic injuries, and is rarely observed in patients with acute poisoning, especially children (1). Children and adults exhibit markedly distinct diagnostic and therapeutic approaches to AOCS. This is the first case of AOCS in children caused by acute herbicide poisoning reported (2). There are no statistical results on the incidence of pediatric acute osteofascial compartment syndrome (PAOCS), and the main cause is trauma, such as fractures (3). Research statistics indicate that the annual incidence of AOCS in women is 0.7 per 100,000, which is one-tenth that in men, and the rate of those requiring DSF is less than 1% (4, 5). DSF is critical and challenging to determine, with leg circumference serving as an important indicator of AOCS tissue pressure. Muscle necrosis in AOCS induces to rhabdomyolysis, even acute renal failure in severe patients (6). In the case report, the diagnosis and treatment of a pediatric patient with AOCS, rhabdomyolysis and even acute renal failure as a result of acute herbicide poisoning. Unlike AOCS of traumatic origin, toxicant-induced AOCS mandates concurrent aggressive detoxification alongside standard limb-salvage measures. Additionally, special attention needs to be paid to the psychological issues of children. We report an exceptional instance of diquat-associated AOCS in a child and chronicle the temporal evolution of disease, decision points at every therapeutic juncture, the urgency and continuity of toxin elimination, and the timing for selecting DSF. These data may serve as a reference for treating herbicide-related AOCS in the pediatric population.

## 2 Case report

The case presents a 13-year-old girl who consumed approximately 60 ml (12 g) of diquat, underwent gastric lavage 10 min post-ingestion, subsequently being transferred to our hospital 17 h later. On arrival the girl was alert and reported that she had initially been asymptomatic immediately after ingestion, but subsequently developed persistent nausea, more than ten episodes of emesis, and profuse diarrhoea. Physical examination revealed a cooperative child with erythematous, oedematous pharyngeal mucosa; no oedema was present in the lower extremities, and the remainder of the systemic survey was unremarkable. Whole-blood qualitative screening confirmed diquat, and quantitative urine diquat concentration was 15,000 ng·ml^−1^. Laboratory data demonstrated hepatic and renal dysfunction. Myoglobin (MB) and creatine kinase-MB (CK-MB) were markedly elevated, indicating acute myocytolysis. Blood indicators were increased, such as C-reactive protein, interleukin 6, white blood cell count and neutrophil granulocyte; peripheral blood smear showed a mild left shift with toxic granulation of neutrophils. Etailed indicators are shown in Table 1. Chest CT revealed bilateral inflammatory infiltrates; ultrasonography and other imaging studies were otherwise normal. Based on the history of massive diquat ingestion, the diagnosis of severe acute diquat poisoning was established. Immediate management included comprehensive gastrointestinal decontamination with concurrent administration of activated charcoal plus mannitol and montmorillonite plus mannitol for adsorption and catharsis; intravenous omeprazole for gastric protection; glutathione plus vitamin C for antioxidant therapy; and high-dose dexamethasone pulse therapy (30 mg daily for 3 days, then tapered by 10 mg daily until discontinuation). On the day of admission extracorporeal toxin removal was initiated: alternating cycles of haemoperfusion and plasma exchange were performed for four consecutive days, complemented by daily CRRT. On hospital days 11 and 19, CRRT was terminated after blood return because the blood pump could not maintain continuous flow. Consequently, the patient received no dialysis sessions on days 11, 20, or 21 of admission.

**Table 1:** Main monitoring indicators.

Monitoring index	Reference range	Day1	Day2	Day4	Day6	Day8	Day10	Day12	Day14	Day21	Day30
MB (ng·ml^−1^)	0–61.50	>2,000	>2,000	>2,000	>2,000		>2,000	1,118	418.80	127.70	124.90
CK (U·L^−1^)	26–140		>8,000	>8,000	6,556.74	5,579.63	892.37	316.60	107.70		
Creatinine (μmol·L^−1^)	46–92	240.86	187.34	133.84	99.30	160.90	173.73	353.81	107.70	579.20	139.60
Blood urea nitrogen (mmol·L^−1^)	2.5–6.1	8.06	9.33	7.22	3.87	7.77	8.70	16.98	6.34	30.02	8.60
Urine volume (ml)		0	0	0	0	0	0	0	0	20	700
C-reactive protein (mg·L^−1^)	≤10	28.24	60.62	180.01	121.50	170.96		191.33	81.46	22.63	24.38
Interleukin 6 (pg·ml^−1^)	<6.60	43.04		173	90.59	123	63.94	73.69		42.84	
White blood cell count (*10^9^·L^−1^)	4.10–11.0	24.61	23.16	8.61	46.45	82.46	72.05	23.42	18.65	8.84	15.26
Neutrophil granulocyte (*10^9^·L^−1^)	1.81–8.30	23.06	21.77	7.44	37.53	69.35	63.26	20.24	15.40	6.69	11.30
Neutrophil-to-lymphocyte ratio	>3.64	49.06	24.74	9.66	7.92	7.58	10.09	12.19	7.59	6.08	4.52
Hemoglobin (g·L^−1^)	114–154	135	145	96	95	75	76	50	85	66	82
CK–MB (ng·ml^−1^)	<3.38	12.60	37.60	83.10		43.10	8.67	2.01	2.80	1.19	5.49
Alanine aminotransferase (U·L^−1^)	0–35	150	333	163		200	162	79		16	
Aspartate aminotransferase (U·L^−1^)	14–36	269.67	681.59	345.63		400.33	386.63	137.65	27.24	18.26	
Lactate dehydrogenase (U·L^−1^)	120–246	1,405	2,504.97	653.10	630	1,210	674.36	381.52	519	239.49	332.70

At 20:54 on the day of admission—21 h post-ingestion-the patient developed cold, cyanotic extremities with mild bilateral lower-limb swelling, increased calf tension and feeble dorsalis pedis pulses. She reported severe bilateral leg pain; the legs were elevated and analgesia was provided with sufentanil. 28 h after ingestion she became delirious and uncooperative, with transient tachycardia that resolved within 10 min. On hospital day 2, physical examination revealed progressive soft-tissue swelling of both lower legs and increased muscular compartment tension; plantar reflexes were absent. All laboratory indices related to AOCS were markedly abnormal (Table 1). Serum CK exceeded 8,000 U·L^−1^, indicating severe muscle necrosis. Ultrasonography of the extremities disclosed circumferential subcutaneous oedema and soft-tissue thickening of both thighs. In conjunction with the patient's clinical presentation and all ancillary investigations-including the triad of agitation, anxiety and inadequate analgesia (“3A” sign)—the multidisciplinary team concluded that severe acute diquat poisoning had precipitated AOCS and rhabdomyolysis, with concomitant multi-organ failure involving the liver, kidneys, myocardium and central nervous system, complicated by systemic inflammatory response syndrome and toxic encephalopathy. Orthopaedic surgery recommended immediate DSF. Although counselled that ongoing compartment hypertension would inexorably worsen the injury, the girl's mother, anxious about possible postoperative sequelae and the long incisional scar that DSF would leave, declined the procedure. Conservative measures were therefore instituted: intravenous mannitol for osmotic diuresis and limb elevation to attenuate oedema. Hospital day 3 was characterised by episodic restlessness and high-pitched screaming that were refractory to incremental doses of analgesics and sedatives.

**Figure 1:**
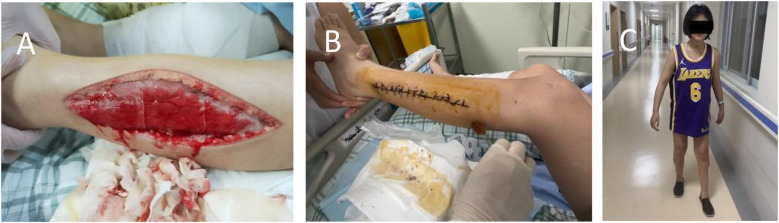
**(A)** Decompressive surgical fasciotomy; **(B)** incisional suture; **(C)** standing photograph of child.

On hospital day 4 the patient became obtunded (Glasgow Coma Scale E3V4M5); both lower extremities were massively swollen, the distal toes were pale, dorsalis pedis pulses were absent, and a violaceous marbling of the overlying skin was evident. Faced with relentless clinical deterioration, the mother consented to emergency bilateral lower-limb DSF. The procedure involved making incisions approximately 20 cm in length, starting from the lower margin of the fibular head on both sides and extending to 6 cm above the tip of the lateral malleolus. The subcutaneous and fascial layers were carefully dissected using a stopover technique. Following DSF surgery, continuous decompression was applied to both lower limbs, with simultaneous monitoring of incisional exudate volume and bilateral lower-limb tension. On hospital day 6, copious serosanguinous drainage persisted from the calf incisions while thigh pressures remained elevated. Dressings were changed and the deep posterior fascial compartments were further released to ensure complete decompression. The main monitoring indicators and leg circumference measurements are detailed in Tables 1, 2, including MB, CK, creatinine, blood urea nitrogen, urine volume and blood indicators, as well as liver enzymes et al. Leucocytosis worsened throughout hospitalisation, attributed to infection and a probable leukemoid reaction. Concurrent critical care included management of respiratory failure, circulatory collapse, toxic encephalopathy, toxic shock syndrome and severe pneumonia caused by carbapenem-resistant Acinetobacter baumannii, complicated by recurrent sepsis and septic shock. Broad-spectrum antimicrobials and supportive therapies were administered accordingly. On hospital day 25, necrotic skin and subcutaneous tissues adjacent to the fasciotomy wounds were debrided and the wounds were closed primarily (Figure 1B). By hospital day 32 renal function had normalised, the fasciotomy wounds had healed satisfactorily and lower-limb motor function had recovered sufficiently to allow unassisted standing (Figure 1C). At two-month follow-up the patient had regained independent ambulation and renal indices remained within reference limits.

**Table 2:** Leg circumferences.

Circumference(cm)	Day1	Day2	Day4	Day6	Day8	Day10	Day12	Day14	Day16
Left thigh	48.5	48.5	52.6	53.0	51.5	49.5	49.5	48.5	45.0
Left lower leg	35.5	37.0	38.7						
Right thigh	48.0	49.0	53.0	52.0	50.0	48.5	50.0	49.0	44.0
Right lower leg	35.5	36.5	38.2						

## 3 Discussion

AOCS is a series of symptoms and signs caused by acute ischemia of muscles and nerves in osteofascial compartment. If it is not treated in time, it can lead to tissue necrosis. Rhabdomyolysis occurs secondary in 12%–44% of patients with compartment syndrome, and acute kidney injury occurs in 10%–55% of patients with rhabdomyolysis (7). Children are more vulnerable to reperfusion injury, acute kidney injury and recurrent compartmental re-stricture; therefore, they require more precise volume management and multimodal analgesia (8). Such patients are often difficult to diagnose and treat early and have poor prognosis. Reports of rhabdomyolysis caused by herbicides are not uncommon. Feng et al. (9) first documented diquat-induced rhabdomyolysis in a 36-year-old woman who ingested 30 ml of diquat; after aggressive extracorporeal elimination therapy, the patient achieved complete clinical recovery and was discharged in stable condition. However, AOCS is rare that it is caused by herbicide poisoning, and no pediatric case. In a systematic review and meta-analysis of PAOCS, fasciotomy was performed in 227 of 233 cases (97.4%). Pain was the most common presenting symptom (88%), followed by paresthesias (32%), and the mean interval from injury to fasciotomy was 25.4 h. Among the documented aetiologies, toxic causes included snake or insect envenomation, drug overdose producing vasotoxic effects (10). A previously reported 2-year-old toddler ingested diquat. During the first 18 h, manifestations were confined to localized signs of intoxication. Systemic toxicity subsequently developed, yet AOCS did not occur. The child succumbed to progressive multi-organ failure 77 h post-exposure (11). We report a case of acute herbicide (diquat) poisoning induced AOCS in a girl with secondary rhabdomyolysis and acute renal failure. The girl underwent necessary DSF due to AOCS and eventually recovered.

PAOCS remains a condition without evidence-based guidelines. Although the underlying pathophysiology mirrors that in adults, developmental differences in anatomy, haemodynamics and pain perception render adult protocols only partially applicable. The classic “5P” signs-pain (or its progression to painless), pallor, pulselessness, paralysis and paresthesia-are frequently appear late in children and are therefore unreliable for early diagnosis. Noonan and McCarthy have proposed the “3A” signs-anxiety, agitation and escalating analgesia-as a more sensitive bedside screen for impending compartmental ischaemia in the pediatric population (12). Clinical history and evolving symptoms remain the cornerstone of diagnosis; indeed, DSF itself is often the de facto confirmatory test (13). Objective confirmation relies on intracompartmental pressure (ICP) monitoring. In adults, an ICP ≥ 30 mmHg is widely accepted as an operative threshold, but normal resting pressures are higher in children and vary anatomically, rendering an adult cut-off inappropriate (14). Consequently, pressure criteria for PAOCS remain controversial. Several laboratory markers correlate with AOCS in adult cohorts: CK > 1,000 U·L^−1^ and elevated myoglobin are consistently associated with muscle necrosis, and serial CK measurements have been proposed as an early diagnostic adjunct. Comparable pediatric data are lacking. Elevated hepatic transaminases and cardiac troponins further reflect global myocyte injury. Inflammatory indices—C-reactive protein, interleukin 6, white blood cell count and neutrophil granulocyte—quantify the systemic inflammatory burden (15, 16). When rhabdomyolysis is present, a neutrophil-to-lymphocyte ratio >3.64 has been validated as an independent predictor of disease severity (17).

Therapeutic approaches to pediatric vs. adult AOCS differ markedly. Current recommendations for fasciotomy in children-encompassing incision technique, underlying pathophysiological rationale, and timing of wound closure-are almost exclusively extrapolated from adult literature. Accumulating evidence, however, demonstrates age-dependent variations in fascial thickness, wound-healing capacity, baseline compartment pressures and post-operative outcomes, underscoring the need for pediatric-specific treatment algorithms rather than direct adoption of adult protocols (18). The American Academy of Orthopaedic Surgeons' guideline on the management of AOCS advises against late fasciotomy in adults when muscle necrosis is established or diagnosis has been delayed >8–12 h (19). By contrast, children possess more robust muscle and skin viability, a wider therapeutic window, and an enhanced reparative capacity; decompression remains indicated irrespective of elapsed time from injury (20). Even in delayed presentations with evident neuromuscular compromise, pediatric patients derive net benefit from fasciotomy, as permanent neurological sequelae are uncommon (21). A recent meta-analysis by Lin et al. (10) found no statistically significant difference in functional outcome between pediatric patients undergoing early vs. late decompression; 88% of children treated >48 h after symptom onset achieved complete recovery. Compared with the extensive incisions typically employed in adults, pediatric practice must also consider psychological sequelae. Multiple small-incision decompressions have been successfully utilised in early AOCS without neurovascular compromise, although their restricted exposure limits application to select cases (22, 23). Adjunctive strategies—including fluorescence-based microangiography for real-time perfusion monitoring, hyperbaric oxygen therapy, and negative-pressure wound therapy prior to delayed primary closure—further enhance wound healing and functional recovery in PAOCS (24).

Diquat is dipyridine herbicide which is some of the most toxic pesticides available, it distribute to most tissues, causing acute renal injury and ultimately, multiorgan failure. Diquat poisoning has no specific antidote, and the mechanism by which diquat causes AOCS remains unclear (25). There is a paucity of pediatric literature on diquat poisoning, and case fatality rates as high as 43% have been reported (11). Chen et al. reported the first successful management of pediatric diquat poisoning complicated by rhabdomyolysis and shock (26). Two additional cases of diquat-induced rhabdomyolysis in adolescents resulted in fatal outcomes (27). The underlying mechanisms of rhabdomyolysis likely involve mitochondrial damage from oxyradical generation and cell membrane disruption via lipid peroxidation, culminating in cellular injury or death. Diquat compromises myocyte membrane integrity through oxidative stress, triggering calcium influx and cellular necrosis. Subsequent systemic inflammatory responses and shock may exacerbate muscular ischemia-hypoxia, establishing a “second-hit” injury model (9, 26–28). Notably, none of these three cases developed AOCS. Rhabdomyolysis and AOCS exhibit a bidirectional causal relationship; when severe, both conditions converge on acute kidney injury as a complication. Other toxic aetiologies of AOCS include snake envenomation, hymenopteran stings and insect bites. A recent systematic review (29) identified 31 such cases, the majority occurring in the pediatric population. Various drugs may also cause AOCS in the patient through local mechanisms including direct cellular toxicity, vasotoxicity or non-traumatic rhabdomyolysis. De Nobili et al. reported three cases of compartment syndrome following intramuscular hydrocarbon injection. When toxic substances injected in a tissue, hydrocarbons can damage microvasculature, leading to liquefactive necrosis. These organic compounds, due to their fat-dissolving properties, can expand and destroy tissue planes, mimicking necrotizing fasciitis. This condition can increase pressure within the tissue fascia, leading to venous hypertension, tissue ischemia, and necrosis (30). In addition, acute compartment syndrome has been reported following opioid epidemic (31), anticoagulant therapy (32), and intramuscular injection of rodenticides (33). AOCS is a surgical emergency, which requires emergent fasciotomy to avoid potentially life—or limb-threatening sequelae. Regardless of the toxic aetiology, the cornerstone of managing PAOCS centres on prompt toxin elimination, targeted supportive care, and emergent fasciotomy. We discuss a pediatric case of acute diquat poisoning resulting in AOCS, emphasizing the seriousness of that highlights the severity of diquat toxicity and the importance of DSF.

## Data Availability

The original contributions presented in the study are included in the article/Supplementary Material, further inquiries can be directed to the corresponding authors.
